# Olfactory Disruption Restructures Collective Behavior and Increases Cohesive Group Dynamics

**DOI:** 10.3390/biology15040360

**Published:** 2026-02-20

**Authors:** Kaihang Chen, Zoe Shteyn, Thomas Ring, Devashish Pande, Joshua Neunuebel

**Affiliations:** 1 Department of Psychological and Brain Sciences, University of Delaware, Newark, DE 19716, USA; chrisch@udel.edu (K.C.); zoex031@gmail.com (Z.S.); tring@udel.edu (T.R.); deva@udel.edu (D.P.); 2 Ross University School of Veterinary Medicine, Basseterre P.O. Box 334, Saint Kitts and Nevis; 3 Interdisciplinary Neuroscience Graduate Program, University of Delaware, Newark, DE 19716, USA; 4 Data Science Institute, University of Delaware, Newark, DE 19713, USA

**Keywords:** collective behavior, olfaction, huddling, social interactions

## Abstract

Olfaction plays a central role in mammalian social behavior, yet its contribution to group coordination remains poorly understood. Here, we show that olfactory impairment in adult C57BL/6J mice (*Mus musculus*) leads to the spontaneous emergence of structured group behavior not observed in controls. Mice with disrupted olfactory input consistently engaged in close-contact interactions that increased over time. We quantified these events and found that aggregation occurred significantly more often than expected by chance, with olfaction-impaired mice exhibiting coordinated dyadic, triadic, and quartet configurations that were spatially enriched, temporally stable, and showed consistent patterns of progression between the states. Unsupervised behavioral modeling revealed that these formations were preceded by structured approach and stationing behaviors. Our findings suggest that olfactory input is a key contributor to maintaining typical social dynamics, and that its absence may lead to altered patterns of group interaction potentially driven by reduced sensory input. This work positions olfaction as a key scaffold for social structure and offers a novel framework for understanding how animals adapt to sensory loss in complex group settings.

## Simple Summary

When adult mice lose their sense of smell, they begin to cluster closely together in ways that differ from typical social behavior. This study used two different methods to disrupt olfactory input and found that the animals consistently formed and maintained close contact over time. These findings suggest that smell is essential for organizing social behavior at the group level. The results highlight how animals adapt when sensory information is compromised.

## 1. Introduction

Group living, widespread across the animal kingdom, gives rise to social behavior [1]. Within groups, animals compete for resources [2], establish dominance hierarchies [3], form affiliative bonds [4,5,6], and, in some species, cooperate to defend territories [7] or raise offspring [8]. These dynamics are not simply additive but emerge from repeated interactions among multiple individuals [9], producing stable patterns such as preferred associations [10] and communal nesting [8,11]. Understanding how such patterns arise has been a central question in behavioral ecology, with research spanning insects [12], birds [8,13], mammals [14], and primates [4,5,7]. While the specific forms of social organization vary, a common principle is that collective behavior emerges from the coordination of individuals acting and reacting to one another over time [15,16]. Identifying the mechanisms that regulate these interactions is therefore essential for linking individual behavior with patterns of social organization in groups.

Coordinated social behavior depends on sensory systems that allow animals to detect, recognize, and respond to conspecifics [17], with visual and auditory cues contributing to courtship, territoriality, parental care, and stress signaling [18,19,20,21], and tactile cues regulating mating and parent–offspring bonding [22,23]. These sensory modalities not only transmit information but also reduce uncertainty, enabling individuals to anticipate and adjust to the actions of others [24]. Among these systems, olfaction is distinctive because chemical cues can be perceived without direct contact and persist after deposition, enabling animals to detect both the immediate presence of conspecifics and individuals that were recently in the area [25,26,27,28]. This persistence makes chemical communication central to structuring interactions across taxa, as seen in pheromone trails that coordinate foraging in ants and scent-marking that shapes territorial boundaries in mammals [28,29]. Despite this recognition, most studies of sensory influences on sociality have focused on pairs of animals interacting in isolation, leaving largely unexplored how specific sensory modalities contribute to the dynamics of groups.

In mammals, olfaction is a major modality of chemical communication and has been linked to diverse forms of individual social behavior [30]. Odors carried in secretions such as urine, saliva, and glandular markings influence mate choice [31,32], parental investment [33], territorial aggression [34], and recognition of conspecifics [35]. Experimental disruption of olfactory input, through surgical removal of the olfactory bulbs [36,37], chemical ablation of the olfactory epithelium [38,39], or genetic knockouts [40,41,42], has consistently revealed impairments in these behaviors. However, the extent of the deficits varies, and in some cases is confounded by collateral effects of the manipulation. Bulbectomy produces severe behavioral changes but also damages connections to central circuits not related to olfactory function [36], while genetic knockouts often carry developmental complications that alter survival and nursing [41]. Chemical impairment of the olfactory epithelium provides a more direct means of reducing sensory input without disturbing brain development, and such studies confirm that olfactory cues are essential for regulating an individual’s social interactions [39,43]. Collectively, this work demonstrates that olfaction is indispensable for many aspects of mammalian social life, but nearly all of this knowledge comes from assays that isolate specific behaviors rather than observing animals in groups.

Far less is known about whether and how olfactory input shapes collective dynamics when multiple animals interact simultaneously. In natural settings, rodents and many other animals live in group contexts in which behaviors overlap, compete, and coalesce into stable patterns [1]. Whether olfaction is required to sustain such organization, or whether groups compensate for its loss through other modalities, remains an open question. To answer this question, we impaired olfaction in mice and monitored mixed-sex groups in a large arena designed to approximate natural conditions, with interactions recorded continuously over extended durations. This approach enabled us to characterize patterns of coordination that emerged in the absence of olfactory cues and to identify a reproducible group-level behavior.

## 2. Materials and Methods

### 2.1. Animals

C57BL/6 mice (6–8 weeks old; Jackson Laboratory, Bar Harbor, ME, USA; strain #000664) were maintained at ~21 °C and 50% humidity under a 12 h light/dark cycle (lights on 7 a.m. to 7 p.m.). Upon arrival from The Jackson Laboratory, mice were singly housed for two weeks prior to behavioral testing to reduce group-housing effects on social behaviors [44]. Each mouse was kept in a ventilated cage containing ALPHA-dri bedding (Animal Specialties and Provisions, LLC; Watertown, TN, USA) and had *ad libitum* access to food and water. All protocols were approved by the University of Delaware Animal Care and Use Committee and conducted in accordance with National Institutes of Health guidelines.

### 2.2. Triton X-100 Nasal Irrigation

To deplete mouse olfactory receptor neurons, intranasal irrigation with Triton X-100 (Thermo Fisher Scientific, Waltham, MA, USA) was performed following an adapted protocol [45]. Mice were anesthetized with 2.5% isoflurane, and a 1 mL syringe fitted with a blunt 30G needle was inserted approximately 2–3 mm into each nostril. Animals received bilateral irrigation with either phosphate-buffered saline (PBS; 100 μL; control) (Thermo Fisher Scientific, Pittsburgh, PA, USA) or 0.7% Triton X-100 dissolved in PBS (100 μL; experimental group). All animals were returned to their home cage and monitored until fully recovered. The experimenter was blinded to the irrigated solution.

### 2.3. Methimazole Injection

As a secondary approach to reduce olfactory function [46], mice received intraperitoneal (*i.p.*) injections of methimazole (MMZ; 100 mg/kg; 2 μL/g dissolved in physiological saline) (Sigma-Aldrich, St. Louis, MO, USA) or vehicle (physiological saline; Phoenix Pharmaceutical, St. Joseph, MO, USA). All animals were returned to their home cage and monitored until fully recovered. The experimenter was blinded to the injected solution.

### 2.4. Group Social Interaction Recording

One day after nasal irrigation or injection, groups of 4 mice (2 males and 2 females) were placed in a large arena (width = 76.2 cm, length = 76.2 cm, height = 60.96 cm) located in an anechoic chamber to reduce external noise and acoustic reflections for social interaction recordings (Triton X-100: *n* = 7 groups, controls: *n* = 7 groups; MMZ: *n* = 4 groups, controls: *n* = 4 groups). The arena comprised extruded aluminum frames (8020, Inc.; Columbia City, IN, USA) surrounded by Sonex foam (Pinta Acoustic, Inc.; Minneapolis, MN, USA; VLW-35) and a mesh-walled cage inside (McMaster-Carr; Robbinsville, NJ, USA; 9318T25). The cage contained ~1.27 cm of ALPHA-dri bedding and was maintained at ~21 °C. Behavior was recorded when mice were in the dark cycle for 5 h (7 p.m. to 12 a.m.), beginning immediately upon introduction of the mice into the arena, using an overhead camera (FLIR GS3-U3-41C6M-C; 30 f/s) controlled with FlyCap2, with infrared lights (GANZ; Cary, NC, USA; IR-LT30) positioned above the cage to ensure clear visualization of the mice. During recordings, mice had *ad libitum* access to food and water (Clear H_2_O; Westbrook, ME, USA) placed at the midpoint of each side of the arena.

Tracking was performed using SLEAP [47]. Briefly, each mouse was labeled with 7 points (nose, left front, right front, left hind, right hind, base of tail, and center of the body). For the Triton X-100 groups and corresponding controls, 1002 frames were labeled and a multi-animal top-down model was trained to predict the instances of each mouse. For the MMZ groups and corresponding controls, 1048 frames were labeled and a multi-animal bottom-up model was trained to predict the instances of each mouse. The first hour of each video was manually checked to confirm identity assignments and positions of the animals. Tracking errors were then manually corrected, and all analyses were performed on the verified, corrected portion of the video. Although animals aggregated at later times, these periods were not analyzed quantitatively because increased physical aggregation reduced tracking reliability.

### 2.5. Buried Food Test

Two days after nasal irrigation or injection, olfactory function was independently assessed using the buried food test [48]. Mice (Triton X-100: *n* = 14, controls: *n* = 13; MMZ: *n* = 6, controls: *n* = 7) were habituated with chocolate sprinkles for 2 consecutive days and then food deprived for 18 h before testing. This cohort of animals was only used for the buried food test. Each mouse was placed in a clean cage (length: 38.1 cm; width: 18.3 cm; height: 13 cm) containing ALPHA-dri bedding with 1.5 g of chocolate sprinkles buried 3 cm in depth at a randomly chosen corner. The behavior of the animals was recorded, and the duration of the search was capped at 300 s to maintain consistency across trials.

### 2.6. Histology

Immediately after behavioral testing, nasal tissue was collected and fixed in 4% PFA (Thermo Fisher Scientific J19943-K2, Tewksbury, MA, USA) overnight at 4 °C. Samples were decalcified in RDO Rapid Decalcifier (Apex Engineering Products Corporation, Sarasota, FL, USA) for 1 h, cryoprotected in 30% sucrose for at least 24 h, and sectioned at 45 µm on a cryostat (Leica CM3050S, Leica Biosystems, Nussloch, Germany). The slices were stained using cresyl violet (Electron Microscopy Sciences 26671-1A, Hatfield, PA, USA) and imaged with a digital camera (World Precision Instruments USBCAM50, Aurora, CO, USA) mounted on a microscope (VWR 89404-890, Aurora, CO, USA) via a coupler (World Precision Instruments 501381, Aurora, CO, USA). For each mouse (Triton X-100: *n* = 11, controls: *n* = 8; MMZ: *n* = 8, controls: *n* = 8), five randomly selected coronal sections along the anterior–posterior axis were analyzed. The regions of interest (ROIs) were defined within anatomically matched portions of olfactory epithelium, and the thickness was measured consistently across all animals. The main olfactory epithelium thickness was measured at each randomly selected ROI using Fiji (ImageJ 1.54f), then an average thickness of olfactory epithelium for each mouse was computed. The experimenter was blinded to the conditions.

### 2.7. Statistical Analyses

Kolmogorov–Smirnov tests and visual inspections were used to determine the normality of the data distribution. For non-normal data, a Wilcoxon rank-sum test was performed. For normally or approximately normally distributed data, a Welch’s *t*-test was performed. To determine whether mice huddled preferentially at specific locations within the arena, the space was divided into sixteen equal regions (four corners, eight edges, and four centers). For each mouse, we calculated the proportion of huddling events that occurred within each region (corners vs. non-corners). These individual proportions were then compared against the expected uniform distribution using a one-sample Wilcoxon signed-rank test. This animal-level summarization reduced dependence among events originating from the same group while allowing assessment of consistent spatial biases across individuals. Statistical tests were performed using R (4.4.1) and Python (3.10).

### 2.8. Hidden Markov Model Validation

Hidden Markov models (HMMs) were fitted to the 5 s pre-huddling behavioral sequences by pooling all huddling events across animals. Because some animals were repeated in different huddling events, this approach does not account for animal-level dependence. However, the number of animals contributing to multiple events was limited, precluding reliable estimation of animal-level random effects within the HMM framework. Thus, we performed an animal-level clustered bootstrap as a sensitivity analysis to evaluate whether our approach affected the HMM. In each bootstrap iteration, animals were resampled with replacement, and all behavioral sequences contributed by each sampled animal were included as a unit. The HMM was refit to each bootstrap sample, and summary metrics describing state persistence were extracted, specifically, we computed stationary-weighted persistence to characterize HMM structure, defined as,

$$\mathrm{wPersistence}=\overset{K}{\underset{s=1}{\sum }}\pi_{s}P_{ss}$$

where *P_ss_* is the self-transition probability for state *s*, π is the stationary distribution of the transition matrix *P*, and *K* is the number of internal states, which in our case, *K* = 2.

## 3. Results

### 3.1. Impairing Olfactory Function in Mice

**Figure 1:**
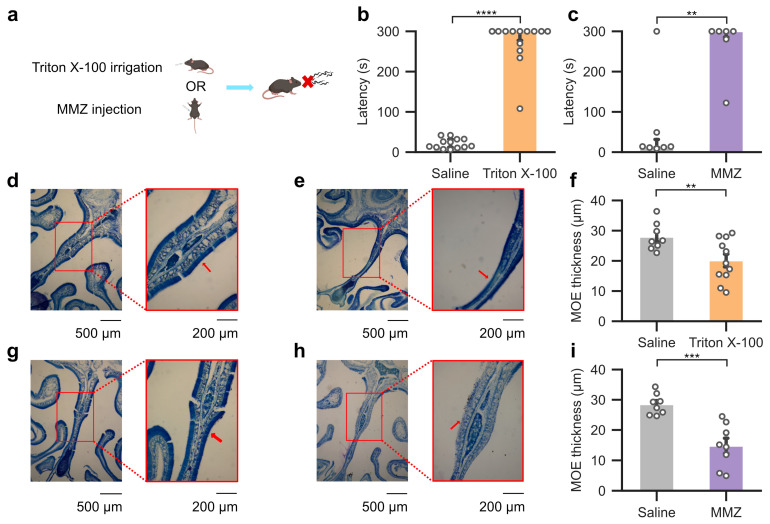
Establishing mouse models with impaired olfactory function. (**a**) Schematic of experimental design showing that mice received either intranasal irrigation with phosphate-buffered saline or 0.7% Triton X-100, or intraperitoneal injection of saline or 100 mg/kg MMZ. (**b**) Performance in the buried food test used to assess olfactory function in mice irrigated with saline (median = 15, IQR = 19.8) or Triton X-100 (median = 300, IQR = 22.5). Plots show the latency (seconds) for individual mice to locate hidden chocolate sprinkles buried in a random location. Trials were capped at 300 s. Wilcoxon rank-sum test, W = 182, *p* < 0.0001, r = 1. (**c**) As in (**b**) but for mice injected with saline (median = 13.8, IQR = 19.8) or MMZ (median = 300, IQR = 14.6). Wilcoxon rank-sum test, W = 38, *p* < 0.01, r = 0.81. (**d**) Representative coronal sections of the nasal cavity from a mouse irrigated with saline. The left image shows an overview of the nasal cavity with a red box marking the region magnified on the right. Red arrows in the magnified image highlight the main olfactory epithelium (MOE). (**e**) As in (**d**) but for a mouse irrigated with Triton X-100. (**f**) Quantification of MOE thickness two days after saline (mean = 27.9, SE = 1.63) and Triton X-100 (mean = 20.1, SE = 2.11) treatment. Each data point represents the average thickness measured across five coronal sections per mouse. Welch’s *t*-test, t = 2.93, *p* < 0.01, d = 1.32. (**g**) As in (**d**) but for a mouse injected with saline. (**h**) As in (**d**) but for a mouse injected with MMZ. (**i**) As in (**f**) but for saline (mean = 28.5, SE = 1.23) and MMZ (mean = 14.8, SE = 2.54) treatment. Welch’s *t*-test, t = 4.85, *p* < 0.001, d = 2.42. **: *p* < 0.01, ***: *p* < 0.001, ****: *p* < 0.0001.

To test how reduced olfactory function influences group social interactions, we first established two models of olfactory impairment. Mice received either binasal irrigation with Triton X-100, which induces reversible injury to the olfactory epithelium through direct damage to olfactory sensory neurons and supporting cells [45,49], or systemic *i.p*. injection of MMZ (Figure 1a), an olfactotoxic agent that causes reversible olfactory epithelial injury primarily through supporting cell damage, resulting in subsequent loss of mature olfactory sensory neurons [50,51,52]. These two distinct, well-established methods both reduce olfactory input [45,46], ensuring that behavioral effects could not be attributed to method-specific artifacts. We confirmed olfactory impairments using both the buried food test [48], a widely used assay of olfactory-guided foraging, and histological analyses [46]. Animals treated with Triton X-100 required significantly more time to locate the food compared to saline irrigated animals (Figure 1b). In 69% of trials with animals that received Triton X-100 (9/13), the 300 s cap was reached without the food being located. Similarly, MMZ injected mice were slower to locate buried food than saline injected controls (Figure 1c), and 67% of trials (4/6) reached the maximum duration without retrieval. Histological analysis corroborated the behavioral results, as OE thickness was significantly reduced after both Triton X-100 (Figure 1d–f) and MMZ treatments (Figure 1g–i). These functional and structural assays were performed in a separate cohort from those used for group social recordings. Together, these results validate two independent approaches for reducing olfactory input. Both methods produced robust sensory impairments, thereby enabling subsequent analysis of how impaired olfaction alters the dynamics of social organization in groups.

### 3.2. Huddling Emerges as a Distinct Pattern of Group Behavior

**Figure 2:**
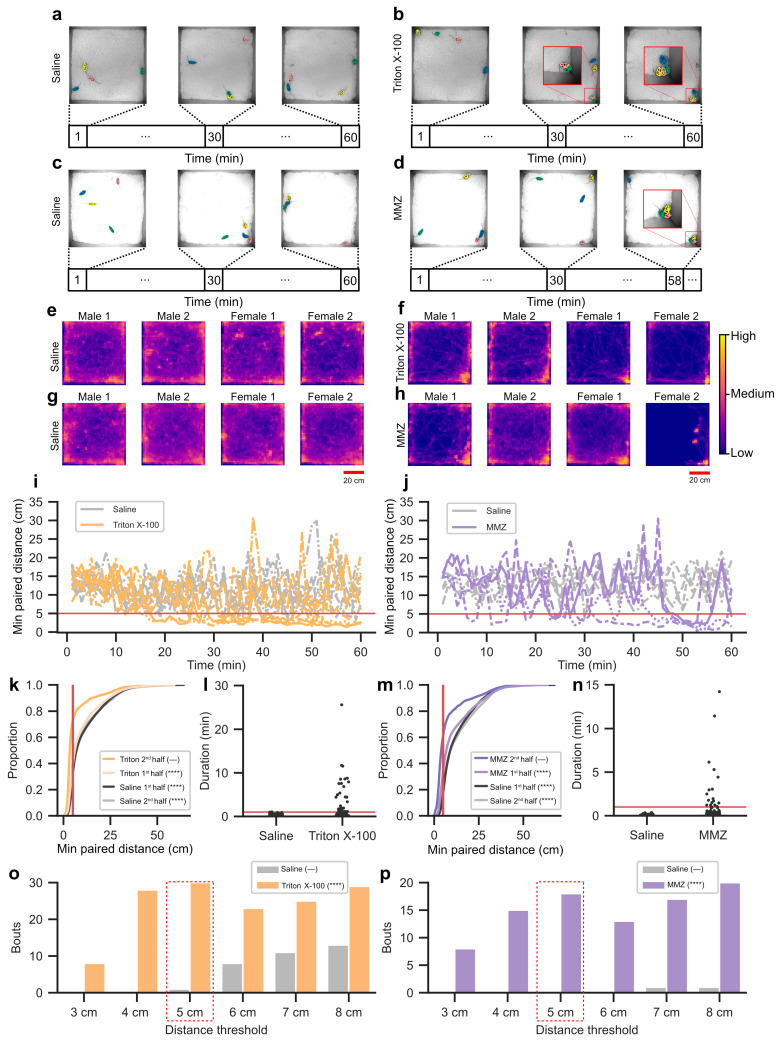
Emergence of huddling behavior in olfactory-impaired mice revealed by spatial and temporal analyses. (**a**) Example images showing a group of saline-irrigated mice interacting during social behavior recording. Images are cropped to the size of the floor of the recording chamber (width = 76.2 cm, length = 76.2 cm). (**b**) As in (**a**) but for mice irrigated with Triton X-100. Red boxes show enlarged view of clustering events. (**c**) As in (**a**) but for saline-injected mice. (**d**) As in (**a**) but for mice injected with MMZ. Red boxes show enlarged view of clustering events. (**e**) Representative occupancy maps showing the spatial distributions of four saline-irrigated mice, with each map corresponding to one animal from the same recording. (**f**) As in (**e**) but for mice irrigated with Triton X-100. (**g**) As in (**e**) but for saline-injected mice. (**h**) As in (**e**) but for MMZ-injected mice. (**i**) Minimum dyadic distance, calculated as the smallest of the six pairwise distances between mice, binned by one-minute intervals across the first hour of the recording. Data are shown for mice irrigated with saline or Triton X-100. The red line marks the 5 cm threshold used as the spatial criterion for sustained proximity. Individual lines correspond to different recordings. (**j**) As in (**i**) but for mice injected with saline or MMZ. (**k**) Minimum dyadic distances for mice irrigated with saline or Triton X-100, shown separately for the first and second halves of the recording. The red line marks the 5 cm threshold used to define as the spatial criterion for sustained proximity. Hyphen (—) indicates the reference group (Triton X-100 2nd half), and all other groups were statistically compared against this reference. Asterisks indicate significance. Triton X-100 1st half vs. 2nd half: Kolmogorov–Smirnov test, D = 0.38, *p* < 0.0001; Triton X-100 2nd half vs. saline 1st half: Kolmogorov–Smirnov test, D = 0.47, *p* < 0.0001; Triton X-100 2nd half vs. saline 2nd half: Kolmogorov–Smirnov test, D = 0.40, *p* < 0.0001. All pairwise tests are corrected with Holm’s method. (**l**) Duration of each event in which the minimum dyadic distance remained below 5 cm for mice treated with PBS or Triton X-100. The red line marks the 1 min threshold used to classify sustained proximity. (**m**) As in (**k**) but for mice treated with saline or MMZ. Hyphen (—) indicates the reference group (MMZ 2nd half), and all other groups were statistically compared against this reference. MMZ 1st half vs. 2nd half: Kolmogorov–Smirnov test, D = 0.30, *p* < 0.0001; MMZ 2nd half vs. saline 1st half: Kolmogorov–Smirnov test, D = 0.45, *p* < 0.0001; MMZ 2nd vs. saline 2nd half: Kolmogorov–Smirnov test, D = 0.42, *p* < 0.0001. All pairwise tests are corrected with Holm’s method. (**n**) As in (**l**), but for mice treated with saline or MMZ. (**o**) Number of sustained proximity events across different minimum dyadic distance thresholds that lasted at least 1 min for groups irrigated with saline or Triton X-100. The dashed red box marks the threshold combination that maximized sustained proximity in Triton X-100 groups while minimizing incidental proximity in saline controls. A Poisson regression model with condition as the predictor and bouts as the response variable showed a significant main effect of condition (β = 1.47, SE = 0.19, z = 7.59, *p* < 0.0001). (**p**) As in (**o**) but for groups injected with saline and MMZ. A Poisson regression model with condition as the predictor and bouts as the response variable showed a significant main effect of condition (β = 3.82, SE = 0.71, z = 5.34, *p* < 0.0001). ****: *p* < 0.0001.

We next asked how olfactory impairment alters group-level organization. Mixed-sex groups of four mice (two males, two females), all receiving the same treatment, were placed in an arena and recorded continuously for five hours, with analyses restricted to the first hour (see Methods). In mice administered Triton X-100, snapshots from one example group illustrate animals dispersed early in the session, shifting by ~30 min to two together while the others remained nearby, and by the end of the hour, all four animals were in close proximity (Figure 2b; Video S1). Mice administered MMZ showed a similar trajectory, with an example sequence from one group showing three animals close together at the end of the first hour (Figure 2d; Video S2). For the control groups, examples show animals distributed at all time points, consistent with continued movement rather than spatial convergence (Figure 2a,b; Videos S3 and S4). Occupancy maps highlighted these differences. Triton X-100 and MMZ groups displayed distinct high-density regions in the arena corners, whereas control groups occupied the arena more uniformly without concentrated regions of activity (Figure 2e–h; Figures S1 and S2).

To operationally define this behavior, we quantified the minimum pairwise distance among the six dyads in each group at every frame and binned these values by one-minute intervals. This analysis showed that minimum pairwise distances in Triton X-100 and MMZ groups declined over the course of the session, whereas distances in their respective saline-irrigated and saline-injected controls remained broadly distributed across the arena (Figure 2i,j; Figures S3 and S4). Cumulative density plots of all frames further highlighted these differences. Triton X-100 groups showed a pronounced shift toward smaller distances in the second half of the session compared to the first and compared to both halves of the saline-irrigated controls (Figure 2k). Similarly, MMZ groups displayed a higher proportion of small distances in the second half (minutes 30–60) compared to their first half (minutes 0–30) and to both halves of saline-injected controls (Figure 2m). Distances less than 5 cm were less frequent in saline-irrigated controls (mean = 36.71%, 95% CI [30.04%, 43.39%]) but more frequent in Triton X-100 groups (mean = 56.18%, 95% CI [38.53%, 73.83%]). Similarly, distances less than 5 cm were less frequent in saline-injected controls (mean = 18.98%, 95% CI [12.01%, 25.96%]) but more frequent in MMZ groups (mean = 47.56%, 95% CI [21.80%, 73.32%]). On this basis, we adopted 5 cm as a data-driven and conservative threshold for identifying close spatial proximity among group members.

To further separate brief encounters from stable organization, we next analyzed the duration of proximity events defined by distances below 5 cm (Figure 2l,n). In saline-irrigated and saline-injected groups, all 13,909 events were short, with only one event exceeding one minute. By contrast, Triton X-100 groups showed 30 events longer than one minute (max = 25.58 min), and MMZ groups showed 18 such events (max = 14.21 min; Figure 2o,p dashed red box). Of these, 17 events persisted for more than two minutes for Triton X-100 groups and 8 events for MMZ groups. Event durations were markedly longer in both impaired conditions than controls. Long-duration events (>1 min) were present in Triton (7/7 groups) and MMZ (4/4 groups) but absent from controls. This clear separation in event durations across conditions motivated the use of one minute as a temporal criterion. Using a 1 min temporal threshold and a 5 cm minimum-distance threshold therefore selectively captured the sustained proximity events unique to olfaction-impaired groups while excluding the brief overlaps that dominated control recordings (Figure 2o,p). Both Triton X-100 irrigated and MMZ injected mice traveled less than saline controls in the first hour of the recording, but maximum speeds did not differ from controls (Figure S5), suggesting although olfactory impaired mice showed reduced locomotor activity, their locomotor ability remained intact. Together, these spatial and temporal criteria identify a reproducible form of group-level behavior, which we term huddling, that emerges specifically under conditions of impaired olfaction.

### 3.3. Quantifying Huddling Behavior

**Figure 3:**
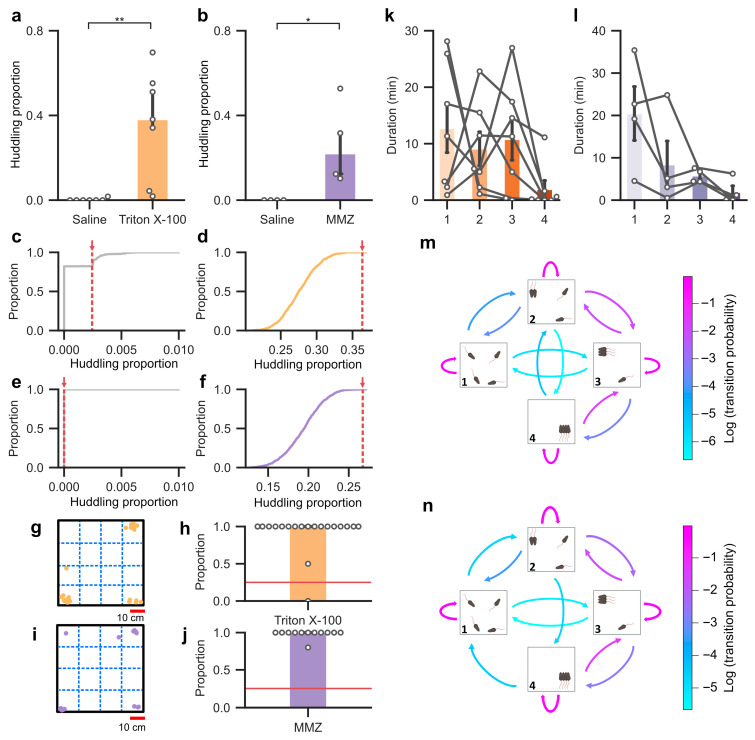
Huddling behavior in olfactory-impaired mice is non-random, spatially localized, and organized into discrete states. (**a**) Proportion of time spent huddling in mice irrigated with saline (median = 0, IQR = 0) or Triton X-100 (median = 0.38, IQR = 0.34). Error bars represent -IQR. Wilcoxon rank-sum test, W = 49, *p* < 0.01, r = 1. (**b**) As in (**a**) but for mice injected with saline (median = 0, IQR = 0) or MMZ (median = 0.22, IQR = 0.25). Wilcoxon rank-sum test, W = 16, *p* < 0.05, r = 1. (**c**) Null distribution of huddling proportions, generated by circularly shifting each animal’s trajectory 1000 times for mice irrigated with saline (*p* = 0.17, two-sided). The dashed red line and arrow mark the observed huddling proportion. (**d**) As in (**c**) but for mice irrigated with Triton X-100 (*p* < 0.01, two-sided). (**e**) As in (**c**) but for mice injected with saline (*p* = 1, two-sided). (**f**) As in (**c**) but for mice injected with MMZ (*p* < 0.01, two-sided). (**g**) Spatial locations of all huddling events pooled across Triton X-100 treated groups, with each dot representing one event. Dashed blue lines indicate region boundaries used to quantify spatial distribution. (**h**) The proportion of huddling in the corners for each individual mouse irrigated with Triton X-100 (median = 1, IQR = 0). Only 1 huddling event was observed in saline controls. Red line indicates the expected probability under a uniform spatial distribution. One sample Wilcoxon signed-rank test, V = 208.5, *p* < 0.0001, r = 0.99. (**i**) As in (**g**) but for MMZ-injected groups. (**j**) As in (**h**) but for MMZ-injected groups (median = 1, IQR = 0). No huddling events were observed in saline controls. One sample Wilcoxon signed-rank test, V = 78, *p* < 0.001, r = 1. (**k**) Proportion of recording time (since first onset huddling event) that Triton X-100 groups spent in different huddling states. State 1 indicates no huddling, state 2 indicates two mice huddling, state 3 indicates three mice huddling, and state 4 indicates all four mice huddling. (**l**) As in (**k**), but for MMZ treated groups. (**m**) Transition probabilities among huddling states in groups of mice irrigated with Triton X-100. (**n**) As in (**m**) but for MMZ-injected groups. *: *p* < 0.05; **: *p* < 0.01.

To assess group differences, we quantified huddling as the fraction of each hour during which mice remained within five centimeters of one another for at least one minute. Because perturbations of olfactory function in rodents can be associated with anxiety-like behavior, which may manifest as increased thigmotaxis in open environments [53], to determine whether these intervals reflect genuine coordination rather than incidental spatial overlap, we compared the observed proportion of time spent huddling in each recording to a permutation-based null distribution generated by circularly shifting each animal’s trajectory 1000 times. This shuffling preserves each animal’s locomotor dynamics but disrupts temporal alignment across individuals, thereby reducing the likelihood of any time-locked coordinated interactions. Huddling was considered above chance when the observed value exceeded 95% of the null distribution (*p* < 0.05). Triton X-100 groups showed significantly higher proportions of time spent huddling than saline-irrigated controls (Figure 3a). MMZ groups also displayed elevated proportions compared to saline-injected controls (Figure 3b). Both treatments exhibited huddling proportions well above their respective shuffled distributions (Figure 3d,f), demonstrating that huddling reflects structured group coordination that exceeds chance expectations. Notably, control groups exhibited significantly less huddling than expected (Figure 3c,e), indicating that they did not converge into sustained group-level huddling states.

Beyond frequency, we examined the spatial distribution of huddling. Both Triton X-100 and MMZ treated mice showed a significant enrichment of huddling in the corners compared to an expected uniform distribution across the arena (Figure 3g–j). To characterize the structure of this behavior, we next defined four distinct huddling states based on spatial configuration and duration. Non-huddling was defined as any time period that did not meet the criteria for huddling. Dyadic huddling was defined as two animals within 5 cm for at least 1 min. Using this criterion, we did not observe any instances in which two dyadic huddling configurations occurred simultaneously within the same group. Triadic huddling was defined as three animals whose mean distance across the three closest pairs was less than 5 cm for at least 1 min. Quartet huddling was defined as four animals forming a compact group, such that the mean of the largest paired distances (i.e., the most separated pairs) was less than 5 cm for at least 1 min. Animals in the Triton X-100 groups frequently entered dyadic, triadic, and quartet states (Figure 3k), and a similar pattern was observed for MMZ-treated groups, which likewise formed dyads, triads, and quartets (Figure 3l). Across huddling states, we did not observe a robust main effect of sex on huddling duration. Although females generally spent more time huddling than males, follow-up analyses revealed a significant sex difference only in the MMZ-treated groups during the dyadic huddling state (Figure S6). Together these analyses show that olfaction-impaired groups not only huddled more often but did so in specific regions of the arena and in larger, well-defined configurations.

To determine whether these huddling states were stable or transient and how groups transitioned among different configurations, we applied a four-state Markov model. Non-huddling, dyad, triad, and quartet served as the four states in the model. Huddling states were defined at the group level and extracted from behavioral recordings at 60 s intervals, yielding discrete-time sequences of huddling configurations for each recording. Each recording contributed one sequence of states, and sequences were concatenated to estimate transition probabilities across recordings. Because saline-irrigated and saline-injected controls seldom formed huddles, models were constructed only for Triton X-100 and MMZ groups. Both models revealed high self-transition probabilities, with within-state values consistently above 70%, indicating that once established, huddling states tended to persist. Transitions between adjacent states (e.g., dyad to triad) were less likely, occurring with probabilities of 2–25%, and rare transitions such as dyad to quartet (state 2 to 4) or triad to non-huddling (state 3 to 1) occurred in less than 1% of cases. This pattern was consistent across both Triton X-100 and MMZ groups (Figure 3m,n). Together, these findings demonstrate that huddling is an organized group process with characteristic spatial preferences and internal dynamics.

### 3.4. Huddling Is Preceded by Distinct Preparatory Behaviors

**Figure 4:**
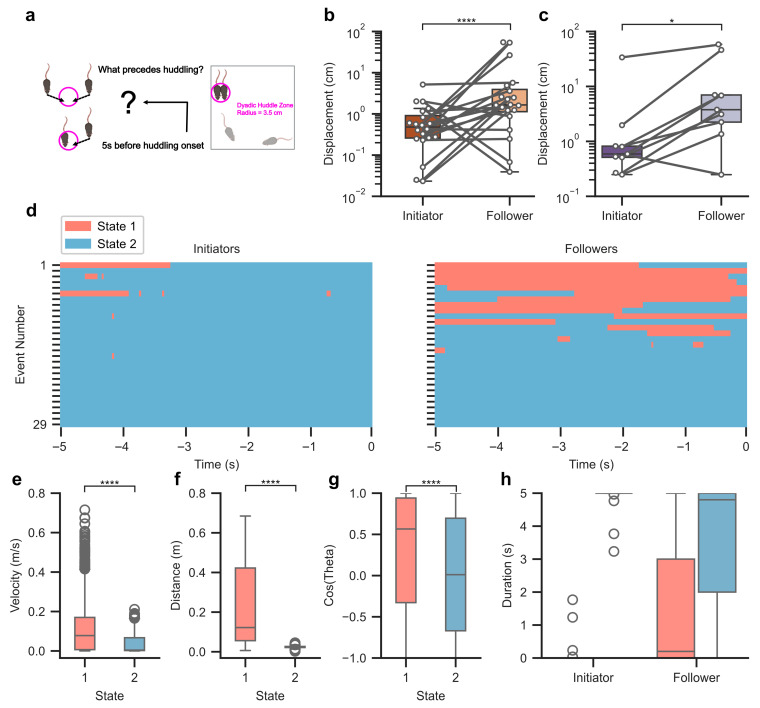
Preparatory movement patterns preceding huddling events revealed by latent-state modeling. (**a**) Schematic illustrating the transition from a non-huddling state to a two-mouse huddling state. (**b**) Displacement of initiator and follower mice during the five seconds preceding the onset of a dyadic huddling event in groups irrigated with Triton X-100. A generalized linear mixed-effects model with a Gamma distribution and log link was fitted because we observed positively skewed residuals. Mouse label (initiator or follower) had a significant effect on displacement (β = 0.72, SE = 0.001, t = 541.4, *p* < 0.0001). Random intercepts for huddling event group and individual identity were included to account for non-independence of observations arising from repeated animals and shared events. (**c**) As in (**b**) but for MMZ-injected groups. A linear mixed-effects model was fitted with mouse label as a fixed effect and both huddling event group and individual identity as random intercepts. The effect of mouse label was significant on displacement (β = 3.76, SE = 0.73, t = 5.2, *p* = 0.016). (**d**) After pooling dyadic huddling events across Triton X-100 and MMZ groups, a two-state Hidden Markov Model identified latent-state sequences for initiators (left) and followers (right) during the five seconds preceding huddling onset. Each row corresponds to one huddling event aligned to onset (time = 0), and rows are ordered by the combined duration that the initiator and follower spent in State 1, with the longest total durations at the top. (**e**) Instantaneous velocity in State 1 and State 2. State 1: median = 0.08 m/s, IQR = 0.16 m/s; State 2: median = 0.004 m/s, IQR = 0.065 m/s; Wilcoxon rank-sum test, W = 7,757,157, *p* < 0.0001, r = 0.53. (**f**) Distance to the huddling center during State 1 and State 2. State 1: median = 0.12 m, IQR = 0.37 m; State 2: median = 0.02 m, IQR = 0.005 m; Wilcoxon rank-sum test, W = 9,997,693, *p* < 0.0001, r = 0.97. (**g**) Head orientation toward the huddling center in State 1 and State 2, represented as the cosine of the angle. State 1: median = 0.57, IQR = 1.27; State 2: median = 0.01, IQR = 1.37; Wilcoxon rank-sum test, W = 6,267,157, *p* < 0.0001, r = 0.23. (**h**) Time spent in the two latent states during the five seconds preceding huddling, shown separately for initiators and followers. *: *p* < 0.05; ****: *p* < 0.0001.

We next investigated the temporal dynamics preceding huddling events, reasoning that if huddling reflects a coordinated social behavior, it should be preceded by consistent patterns. To do so, we identified initiators and followers for each two-mouse huddling state based on the precise moment each mouse entered the huddling circle (Figure 4a). Entry was defined as the time in which the mouse’s distance to the huddling center, computed as the geometric center of all mice involved in the impending huddle, became less than or equal to a 3.5 cm radius and remained within that radius for at least 1 s. The mouse that entered first was labeled the initiator, and the second was labeled the follower. In both Triton X-100 and MMZ groups, followers exhibited greater displacement, measured as change in position from start to end point during the five seconds preceding huddling. For Triton X-100 groups, followers traveled significantly more with a median of 1.65 cm (IQR: 2.79 cm) compared to 0.48 cm (IQR: 0.65 cm) for initiators (Figure 4b). For MMZ groups, followers also traveled significantly more with a median of 4.75 cm (IQR: 3.77 cm) compared to 0.59 cm (IQR: 0.31 cm) for initiators (Figure 4c). These results suggest that followers were more active in the moments leading up to huddling.

To capture finer-grained structure in pre-huddling movements, we pooled dyadic huddling events from Triton X-100 and MMZ groups after confirming that the two treatments did not differ in pre-huddling displacement for initiators (Wilcoxon rank-sum test, W = 72, *p* = 0.42) or followers (W = 58, *p* = 0.14). This yielded 29 events, with 150 frames per event (8,700 total time points) corresponding to the five seconds preceding huddling onset. Using instantaneous speed and distance to the huddling center as observable features, we fitted Hidden Markov Models with 2–4 latent states to this pooled dataset. Model selection using Akaike Information Criterion and Bayesian Information Criterion identified the two-state model as the most parsimonious. The original pooled HMM exhibited high temporal stability, with a stationary-weighted persistence of 0.996 (i.e., the probability of self-transition for each latent state weighted by that state’s stationary occupancy). In the animal-level clustered bootstrap analysis, stationary-weighted persistence was highly consistent across resampled datasets (median = 0.996; 95% bootstrap CI: [0.992, 0.998]). The bootstrap distribution closely overlapped the estimate from the pooled model, indicating that the inferred HMM structure was robust to accounting for animal-level dependence. Raster plots of the inferred state assignments (Figure 4d) revealed structured alternations between latent states prior to huddling onset, indicating consistent motifs in pre-huddling behavior. The latent states were interpreted post hoc based on their kinematic and positional signatures, which showed clear differences in velocity (Figure 4e), distance from the huddling center (Figure 4f), and directional orientation (Figure 4g). In State 1, animals moved at higher velocity, were located farther from the huddling center, and displayed stronger directional orientation toward it, whereas in State 2, animals moved more slowly, remained close to the center, and showed weaker directional alignment. Based on these features, we refer to the first state as the approaching state and the second state as the stationing state.

To examine the distribution of the states across roles, we used Gamma generalized linear mixed-effects models with log links where mouse label and state served as fixed effects and both huddling event and animal served as random effects (Figure 4h). The main effect of state was significant (β = 3.76, SE = 0.60, t = 6.27, *p* < 0.0001), suggesting mice generally spent more time stationing than approaching in the five seconds before huddling. However, the interaction between state and role was also significant (β = 2.90, SE = 0.85, t = 3.42, *p* < 0.001). Post hoc pairwise comparisons of model-estimated marginal means with Holm’s method revealed that the expected duration of approaching was substantially lower in initiators compared to followers (ratio = 0.077, z = −4.28, *p* < 0.001), indicating that followers exhibited approximately 13-fold longer time approaching. To quantify whether pre-huddling behavior predicted which mouse became the initiator or follower, we computed for each animal the ratio of time spent in the approaching state to time spent in the stationing state during the five seconds before huddling. This ratio was log-transformed to normalize its distribution, and the resulting value was used as a predictor in a logistic regression model with role (initiator versus follower) as the binary outcome. The model included individual animals as random intercepts. The model revealed a significant positive effect of the log-transformed approach-to-stationing ratio (β = 0.136, SE = 0.044, z = 3.10, *p* < 0.01), indicating that animals that spent relatively more time approaching were more likely to be labeled as followers. This pattern indicates that the active approach into a huddle was specific to followers, while initiators tended to stay in place once they had occupied the huddling location.

## 4. Discussion

This study reveals that olfactory impairment in adult mice triggers a previously undescribed form of group coordination, sustained huddling behavior. Using two distinct methods, Triton X-100 nasal irrigation and MMZ injection, we found that mice consistently aggregated in close proximity and maintained these groupings over extended periods, especially in corners. This behavior was absent in controls, emerged gradually over the first hour of interaction, and displayed distinct internal dynamics, including preparatory states uncovered through hidden Markov modeling. Together, these results demonstrate that olfactory input plays a critical role in regulating not just dyadic interactions, but the broader architecture of group behavior in mammals.

Triton X-100 and MMZ are widely used models of olfactory epithelial injury and were selected here to perturb olfactory function during social interaction. Consistent with prior work [54,55], mice treated with either manipulation showed significant impairment in finding buried food compared to controls, indicating reduced olfactory function. However, a subset of treated animals (Triton X-100, *n* = 4; MMZ, *n* = 2) successfully located the buried food at two days post-treatment, suggesting that olfactory sensory neuron ablation was incomplete in at least some individuals. While variability in olfactory impairment is expected following intranasal Triton X-100 administration, the observation that some MMZ-treated mice successfully performed the buried food task was unexpected and indicates residual olfactory function in those animals at the time of testing. Importantly, despite this residual olfactory function in a subset of animals, robust and consistent changes in group-level social behavior were observed across treatment conditions. These findings indicate that partial disruption of olfactory input is sufficient to alter collective social organization and promote huddling behavior, and that the observed phenotype does not require complete loss of olfactory sensory neurons.

The emergence of huddling in olfaction-impaired mice underscores the broader role of olfaction cues in structuring social organization. In social species, coordinated behaviors depend heavily on sensory cues that guide interaction timing, location, and partner choice [56]. While vision and audition have been well-characterized in these roles [18,19,20,21], olfaction offers unique advantages due to its persistence and spatial diffusion, enabling detection of both current and recent presence of conspecifics [57,58,59,60]. Disruption of olfactory signaling has been shown to impair maternal care [33], mating [61], and territoriality [62], though these studies often focus on dyadic interactions. Our work extends this literature by revealing that olfactory loss alters group-level dynamics, resulting in spatial convergence and temporally organized aggregation. The preparatory behaviors preceding huddling, such as coordinated approaches and stationing, suggest that mice, even with compromised sensory input, retain the capacity to form structured social patterns. These findings align with broader models of collective animal behavior, where group-level organization can emerge from individuals following simple rules and adjusting their behavior in response to local cues [63,64,65].

One interpretation of this phenomenon is that huddling reflects a defensive adaptation to sensory uncertainty. Olfactory loss removes a critical modality for detecting conspecifics [66] and potential threats [67], increasing environmental ambiguity and perceived risk. In natural settings, animals often respond to uncertainty or danger by seeking group cohesion, which can improve survival through thermoregulatory and energetic benefits [68]. From an evolutionary perspective, peripheral individuals in a group are more vulnerable to predation, a pattern documented across vertebrates and invertebrates [69,70,71,72]. The tendency to aggregate in corners, which provide physical boundaries, may enhance this strategy by limiting potential avenues of approach. By moving closer to others and positioning themselves near walls, mice may effectively reduce their vulnerability and improve their odds of avoiding attack, even in the absence of an actual predator. Such behaviors can arise from simple, non-cognitive rules like approaching conspecifics [73] or exhibiting anxiety-like behaviors [74] and may be more prominent under conditions of sensory deprivation that limit environmental assessment. Thus, huddling may emerge as an evolutionarily conserved response to increased uncertainty, modulated by the availability of sensory inputs that inform risk assessment.

Alternatively, huddling may serve as a prosocial compensatory mechanism driven by the disruption of olfactory-mediated affiliative behaviors. Olfaction is essential for social recognition and emotional regulation in rodents [75], and its disruption has been associated with anxiety-like and depressive phenotypes, though effects on anxiety can vary across paradigms [74], suggesting a role in maintaining socio-emotional homeostasis, the balance of internal emotional states through social interaction [76]. In rodents, affiliative contact and group housing can buffer stress responses [75,77], and the increased physical proximity we observed may represent a compensatory strategy for restoring social connectedness through tactile or thermal cues. The approach behavior preceding huddling implies active engagement rather than passive convergence, consistent with affiliative drives under stress [75]. Studies in primates have shown that animals under chronic stress increase huddling and reduce exploratory behavior [78], supporting the idea that huddling can serve as a comfort-seeking response. While we did not directly measure stress or affective state, previous work has shown that social contact can alleviate behavioral and neural markers of distress in rodents [75]. Future work should examine whether the huddling we observed in olfactory-impaired mice serves a similar stress-buffering function, or simply reflects a shift in sensory priorities under deprivation.

A third possibility is that huddling reflects a thermoregulatory drive, a mechanism by which rodents reduce heat loss through social aggregation [79]. This behavior is well established in neonates [66] and persists in adults when exposed to cold or sub-thermoneutral conditions [80]. Recent studies have shown that adult mice increase huddling and exhibit lower body temperatures under cool ambient conditions [81], and computational models suggest that such behavior can emerge from local thermal feedback and interaction rules without requiring social cognition [73]. Although our study maintained constant ambient temperature, standard laboratory settings are often below the murine thermoneutral zone [81] and may impose mild thermal demands. This mild but persistent thermal stress could interact with developmental factors, as studies show that huddling patterns can be shaped by prior experience and local feedback [66,73], indicating that thermoregulatory behavior may reflect both physical needs and learned strategies. However, the specificity of the behavior to olfactory loss and its structured emergence argue against a purely thermoregulatory mechanism. It is more likely that thermal factors contribute alongside sensory and social influences to produce the observed group convergence.

Importantly, the consistency of huddling behavior across two distinct experimental approaches to olfactory disruption, MMZ and Triton X-100, reinforces the conclusion that olfactory input is a key regulator of group-level social organization. Both manipulations induce reversible injury to the olfactory epithelium, with basal cells spared and subsequent regeneration of olfactory sensory neurons but differ in their initial cellular targets [45,51,52]. Triton X-100 acts through direct epithelial damage affecting olfactory sensory neurons and supporting cells [45], whereas MMZ induces olfactory epithelial injury that results in subsequent loss of mature olfactory sensory neurons [50,51,82,83]. The observation that huddling emerges under both conditions strengthens the interpretation that this behavior reflects a specific consequence of olfactory disruption rather than nonspecific effects of epithelial injury, inflammation, or illness. The use of multiple approaches targeting the same sensory modality therefore enhances internal validity and supports the robustness of the observed behavioral phenotype.

Finally, these findings raise broader questions about how multisensory integration supports collective behavior. Social coordination in mammals relies on the interplay among olfactory, auditory, visual, and thermal signals [66,79,80,84,85]. The emergence of structured group behavior following olfactory disruption suggests that collective dynamics are shaped not only by external cues, but also by how animals adaptively prioritize reliable sensory inputs under uncertainty. In such conditions, animals are thought to reweight sensory modalities to guide social decisions [66,86], an adaptive strategy observed in both social and nonsocial behaviors. To further clarify these mechanisms, future work should incorporate multimodal tracking and targeted sensory disruptions to dissect the relative contributions of each modality. Ultimately, these results highlight the role of olfaction in scaffolding social structure and point to the broader importance of sensory redundancy in maintaining cohesion within animal groups.

## 5. Conclusions

Our findings uncover a novel influence of olfactory processing on the structure and coordination of group behavior in mice. When olfactory input was disrupted, animals exhibited a robust increase in huddling, which was marked by prolonged periods of mice in close proximity and stable group configurations. Huddling occurred in spatially defined patterns and emerged consistently over time, distinguishing them from incidental and transient interactions. Computational analysis of the time preceding huddling revealed organized sequences of behavior, including directed approaches and sustained positioning, suggesting that these group states may arise through structured, anticipatory coordination. The replication of this pattern across two distinct models of olfactory impairment strengthens the conclusion that olfactory input is critical for regulating group-level social organization. When olfactory input is absent, mice adopt alternative group behaviors such as prolonged physical proximity that help maintain coordinated social structure despite reduced sensory information. This work highlights olfaction as a key sensory scaffold in group dynamics and opens new avenues for studying how animals adapt to sensory deficits within social contexts.

## Data Availability

Data and code uploaded to OSF, https://osf.io/v5zpy/overview (accessed on 21 December 2025).

## Supplementary Materials

The following supporting information can be downloaded at: https://www.mdpi.com/article/10.3390/biology15040360/s1, Figure S1: Spatial analysis of individual animal occupancy for Triton X-100 experiments; Figure S2: Spatial analysis of individual animal occupancy for MMZ experiments; Figure S3: Minimum dyadic distances for Triton X-100 experiments; Figure S4: Minimum dyadic distances for MMZ experiments; Figure S5: Olfactory-impaired mice showed reduced locomotor activities, but not ability to move; Figure S6: Both male and female olfactory-impaired mice exhibit huddling behavior; Video S1: Triton X-100 irrigated mice example; Video S2: MMZ injected mice example; Video S3: Saline irrigated mice example; Video S4: Saline injected mice example.

## Abbreviations

The following abbreviations are used in this manuscript:

MMZ	Methimazole
PFA	Paraformaldehyde
HMM	Hidden Markov model
IQR	Inter-quartile range
SE	Standard error
ROI	Region of interest
CI	Confidence intervals

## References

[1] Krause J., Ruxton G.D. (2002). Living in Groups.

[2] Smolla M., Gilman R.T., Galla T., Shultz S. (2015). Competition for resources can explain patterns of social and individual learning in nature. Proc. R. Soc. B Biol. Sci..

[3] Tibbetts E.A., Pardo-Sanchez J., Weise C. (2022). The establishment and maintenance of dominance hierarchies. Philos. Trans. R. Soc. B Biol. Sci..

[4] Silk J.B., Beehner J.C., Bergman T.J., Crockford C., Engh A.L., Moscovice L.R., Wittig R.M., Seyfarth R.M., Cheney D.L. (2010). Strong and Consistent Social Bonds Enhance the Longevity of Female Baboons. Curr. Biol..

[5] Silk J.B., Beehner J.C., Bergman T.J., Crockford C., Engh A.L., Moscovice L.R., Wittig R.M., Seyfarth R.M., Cheney D.L. (2009). The benefits of social capital: Close social bonds among female baboons enhance offspring survival. Proc. R. Soc. B Biol. Sci..

[6] Thompson N.A., Cords M. (2018). Stronger social bonds do not always predict greater longevity in a gregarious primate. Ecol. Evol..

[7] Micheletta J., Waller B.M., Panggur M.R., Neumann C., Duboscq J., Agil M., Engelhardt A. (2012). Social bonds affect anti-predator behaviour in a tolerant species of macaque, Macaca nigra. Proc. R. Soc. B Biol. Sci..

[8] Griesser M., Drobniak S.M., Nakagawa S., Botero C.A. (2017). Family living sets the stage for cooperative breeding and ecological resilience in birds. PLoS Biol..

[9] Couzin I.D. (2009). Collective cognition in animal groups. Trends Cogn. Sci..

[10] Jowett S.L., Barker Z.E., Amory J.R. (2023). Preferential associations in an unstable social network: Applying social network analysis to a dynamic sow herd. Front. Vet. Sci..

[11] Ruch J., Herberstein M.E., Schneider J.M. (2014). Families hunt more successfully: Effect of group composition on hunting and communal feeding. Anim. Behav..

[12] Ferreira C.H., Moita M.A. (2020). Behavioral and neuronal underpinnings of safety in numbers in fruit flies. Nat. Commun..

[13] Johnson A.E., Masco C., Pruett-Jones S. (2018). Song recognition and heterospecific associations between 2 fairy-wren species (Maluridae). Behav. Ecol..

[14] Edwards D.A., Roberts R.L. (1972). Olfactory bulb removal produces a selective deficit in behavioral thermoregulation. Physiol. Behav..

[15] Ioannou C.C., Laskowski K.L. (2023). A multi-scale review of the dynamics of collective behaviour: From rapid responses to ontogeny and evolution. Philos. Trans. R. Soc. B Biol. Sci..

[16] Fisher D.N., Ilany A., Silk M.J., Tregenza T. (2017). Analysing animal social network dynamics: The potential of stochastic actor-oriented models. J. Anim. Ecol..

[17] Chen P., Hong W. (2018). Neural Circuit Mechanisms of Social Behavior. Neuron.

[18] Strasser S., Dixon A.K. (1986). Effects of visual and acoustic deprivation on agonistic behaviour of the albino mouse (*M. musculus* L.). Physiol. Behav..

[19] Neunuebel J.P., Taylor A.L., Arthur B.J., Egnor S.E.R. (2015). Female mice ultrasonically interact with males during courtship displays. eLife.

[20] Hofer M.A. (1996). Multiple regulators of ultrasonic vocalization in the infant rat. Psychoneuroendocrinology.

[21] Liu H.-X., Lopatina O., Higashida C., Fujimoto H., Akther S., Inzhutova A., Liang M., Zhong J., Tsuji T., Yoshihara T. (2013). Displays of paternal mouse pup retrieval following communicative interaction with maternal mates. Nat. Commun..

[22] Contreras J.L., Agmo A. (1993). Sensory control of the male rat’s copulatory thrusting patterns. Behav. Neural Biol..

[23] Champagne F.A., Curley J.P. (2005). How social experiences influence the brain. Curr. Opin. Neurobiol..

[24] Randolet J., Lucas J.R., Fernández-Juricic E., Foster S. (2014). Non-Redundant Social Information Use in Avian Flocks with Multisensory Stimuli. Ethology.

[25] Li Y., Dulac C. (2018). Neural coding of sex-specific social information in the mouse brain. Curr. Opin. Neurobiol..

[26] Boesveldt S., Parma V. (2021). The importance of the olfactory system in human well-being, through nutrition and social behavior. Cell Tissue Res..

[27] Ryan B.C., Young N.B., Moy S.S., Crawley J.N. (2008). Olfactory cues are sufficient to elicit social approach behaviors but not social transmission of food preference in C57BL/6J mice. Behav. Brain Res..

[28] Roberts S.C. (2007). Scent marking. Rodent Societies: An Ecological and Evolutionary Perspective.

[29] Jackson D.E., Martin S.J., Ratnieks F.L.W., Holcombe M. (2007). Spatial and temporal variation in pheromone composition of ant foraging trails. Behav. Ecol..

[30] Barabas A.J., Dijak S.R., Yatcilla J.F., Walker D.N., Gaskill B.N. (2021). Modulating captive mammalian social behavior: A scoping review on olfactory treatments. Appl. Anim. Behav. Sci..

[31] Roberts S.A., Simpson D.M., Armstrong S.D., Davidson A.J., Robertson D.H., McLean L., Beynon R.J., Hurst J.L. (2010). Darcin: A male pheromone that stimulates female memory and sexual attraction to an individual male’s odour. BMC Biol..

[32] Harmeier A., Meyer C.A., Staempfli A., Casagrande F., Petrinovic M.M., Zhang Y.P., Kunnecke B., Iglesias A., Honer O.P., Hoener M.C. (2018). How Female Mice Attract Males: A Urinary Volatile Amine Activates a Trace Amine-Associated Receptor That Induces Male Sexual Interest. Front. Pharmacol..

[33] Levy F., Keller M., Poindron P. (2004). Olfactory regulation of maternal behavior in mammals. Horm. Behav..

[34] Ralls K. (1971). Mammalian scent marking. Science.

[35] Bowers J.M., Alexander B.K. (1967). Mice: Individual recognition by olfactory cues. Science.

[36] Edwards D.A., Burge K.G. (1973). Olfactory control of the sexual behavior of male and female mice. Physiol. Behav..

[37] Liebenauer L. (1996). Social Organization and Aggression in a Group of Olfactory Bulbectomized Male Mice. Physiol. Behav..

[38] Bean N.J. (1982). Modulation of agonistic behavior by the dual olfactory system in male mice. Physiol. Behav..

[39] Keller M., Douhard Q., Baum M.J., Bakker J. (2006). Destruction of the main olfactory epithelium reduces female sexual behavior and olfactory investigation in female mice. Chem. Senses.

[40] Mandiyan V.S., Coats J.K., Shah N.M. (2005). Deficits in sexual and aggressive behaviors in Cnga2 mutant mice. Nat. Neurosci..

[41] Matsuo T., Hattori T., Asaba A., Inoue N., Kanomata N., Kikusui T., Kobayakawa R., Kobayakawa K. (2015). Genetic dissection of pheromone processing reveals main olfactory system-mediated social behaviors in mice. Proc. Natl. Acad. Sci. USA.

[42] Wang Z., Storm D.R. (2011). Maternal behavior is impaired in female mice lacking type 3 adenylyl cyclase. Neuropsychopharmacology.

[43] Zhang Y.F., Janke E., Bhattarai J.P., Wesson D.W., Ma M. (2022). Self-directed orofacial grooming promotes social attraction in mice via chemosensory communication. iScience.

[44] Jones R.B., Nowell N.W. (2012). Aversive potency of urine from dominant and subordinate male laboratory mice (*Mus musculus*): Resolution of a conflict. Aggress. Behav..

[45] Cummings D.M., Emge D.K., Small S.L., Margolis F.L. (2000). Pattern of olfactory bulb innervation returns after recovery from reversible peripheral deafferentation. J. Comp. Neurol..

[46] Haglin S., Bohm S., Berghard A. (2021). Single or Repeated Ablation of Mouse Olfactory Epithelium by Methimazole. Bio Protoc..

[47] Pereira T.D., Aldarondo D.E., Willmore L., Kislin M., Wang S.S.H., Murthy M., Shaevitz J.W. (2019). Fast animal pose estimation using deep neural networks. Nat. Methods.

[48] Yang M., Crawley J.N. (2009). Simple behavioral assessment of mouse olfaction. Curr. Protoc. Neurosci..

[49] Iqbal T., Byrd-Jacobs C. (2010). Rapid degeneration and regeneration of the zebrafish olfactory epithelium after triton X-100 application. Chem. Senses.

[50] Suzukawa K., Kondo K., Kanaya K., Sakamoto T., Watanabe K., Ushio M., Kaga K., Yamasoba T. (2011). Age-related changes of the regeneration mode in the mouse peripheral olfactory system following olfactotoxic drug methimazole-induced damage. J. Comp. Neurol..

[51] Bergstrom U., Giovanetti A., Piras E., Brittebo E.B. (2003). Methimazole-induced damage in the olfactory mucosa: Effects on ultrastructure and glutathione levels. Toxicol. Pathol..

[52] Xie F., Zhou X., Genter M.B., Behr M., Gu J., Ding X. (2011). The tissue-specific toxicity of methimazole in the mouse olfactory mucosa is partly mediated through target-tissue metabolic activation by CYP2A5. Drug Metab. Dispos..

[53] Glinka M.E., Samuels B.A., Diodato A., Teillon J., Feng Mei D., Shykind B.M., Hen R., Fleischmann A. (2012). Olfactory deficits cause anxiety-like behaviors in mice. J. Neurosci..

[54] Mast T.G., Zuk K., Rinke A., Quasem K., Savard B., Brobbey C., Reiss J., Dryden M. (2019). Temporary Anosmia in Mice Following Nasal Lavage with Dilute Detergent Solution. Chem. Senses.

[55] Kim J., Choi Y., Ahn M., Ekanayake P., Tanaka A., Matsuda H., Shin T. (2019). Microglial and astroglial reaction in the olfactory bulb of mice after Triton X-100 application. Acta Histochem..

[56] Wasserman S.M., Frye M.A. (2015). Group behavior: Social context modulates behavioral responses to sensory stimuli. Curr. Biol..

[57] Blanchard D.C., Griebel G., Blanchard R.J. (2003). The Mouse Defense Test Battery: Pharmacological and behavioral assays for anxiety and panic. Eur. J. Pharmacol..

[58] Hurst J.L., Beynon R.J. (2004). Scent wars: The chemobiology of competitive signalling in mice. Bioessays.

[59] Eisenberg J.F. (1981). The Mammalian Radiations: An Analysis of Trends in Evolution, Adaptation, and Behavior.

[60] Brown R.E., Macdonald D.W. (1985). Social Odours in Mammals.

[61] Decoster L., Trova S., Zucca S., Bulk J., Gouveia A., Ternier G., Lhomme T., Legrand A., Gallet S., Boehm U. (2024). A GnRH neuronal population in the olfactory bulb translates socially relevant odors into reproductive behavior in male mice. Nat. Neurosci..

[62] Nakamura K., Kikusui T., Takeuchi Y., Mori Y. (2007). The critical role of familiar urine odor in diminishing territorial aggression toward a castrated intruder in mice. Physiol. Behav..

[63] Couzin I.D., Krause J., James R., Ruxton G.D., Franks N.R. (2002). Collective memory and spatial sorting in animal groups. J. Theor. Biol..

[64] Amichay G., Nagy M. (2025). On the integration of collective motion and temporal synchrony in animal collectives. Mov. Ecol..

[65] Sato D.X., Takahashi Y. (2025). Neurogenomic and behavioral principles shape freezing dynamics and synergistic performance in Drosophila melanogaster. Nat. Commun..

[66] Alberts J.R., Brunjes P.C. (1978). Ontogeny of thermal and olfactory determinants of huddling in the rat. J. Comp. Physiol. Psychol..

[67] Blanco-Hernandez E., Valle-Leija P., Zomosa-Signoret V., Drucker-Colin R., Vidaltamayo R. (2012). Odor memory stability after reinnervation of the olfactory bulb. PLoS ONE.

[68] Ruf T., Bieber C. (2020). Use of social thermoregulation fluctuates with mast seeding and reproduction in a pulsed resource consumer. Oecologia.

[69] Quinn J.L., Cresswell W. (2006). Testing domains of danger in the selfish herd: Sparrowhawks target widely spaced redshanks in flocks. Proc. R. Soc. B Biol. Sci..

[70] De Vos A., O’Riain M.J. (2009). Sharks shape the geometry of a selfish seal herd: Experimental evidence from seal decoys. Biol. Lett..

[71] Bumann D., Krause J., Rubenstein D. (1997). Mortality Risk of Spatial Positions in Animal Groups: The Danger of Being in the Front. Behaviour.

[72] Rayor L.S., Uetz G.W. (1990). Trade-offs in foraging success and predation risk with spatial position in colonial spiders. Behav. Ecol. Sociobiol..

[73] Schank J.C., Alberts J.R. (1997). Self-Organized Huddles of Rat Pups Modeled by Simple Rules of Individual Behavior. J. Theor. Biol..

[74] Ahn S., Choi M., Kim H., Yang E.J., Mahmood U., Kang S.I., Shin H.W., Kim D.W., Kim H.S. (2018). Transient Anosmia Induces Depressive-like and Anxiolytic-like Behavior and Reduces Amygdalar Corticotropin-Releasing Hormone in a ZnSO_4_-Induced Mouse Model. Chem. Senses.

[75] Kiyokawa Y., Takeuchi Y., Nishihara M., Mori Y. (2009). Main olfactory system mediates social buffering of conditioned fear responses in male rats. Eur. J. Neurosci..

[76] Kikusui T., Winslow J.T., Mori Y. (2006). Social buffering: Relief from stress and anxiety. Philos. Trans. R. Soc. Lond. B Biol. Sci..

[77] Hennessy M.B., Kaiser S., Sachser N. (2009). Social buffering of the stress response: Diversity, mechanisms, and functions. Front. Neuroendocr..

[78] Qin D.D., Rizak J., Feng X.L., Yang S.C., Lu L.B., Pan L., Yin Y., Hu X.T. (2016). Prolonged secretion of cortisol as a possible mechanism underlying stress and depressive behaviour. Sci. Rep..

[79] Harshaw C., Alberts J.R. (2012). Group and individual regulation of physiology and behavior: A behavioral, thermographic, and acoustic study of mouse development. Physiol. Behav..

[80] Sukhchuluun G., Zhang X.Y., Chi Q.S., Wang D.H. (2018). Huddling Conserves Energy, Decreases Core Body Temperature, but Increases Activity in Brandt’s Voles (*Lasiopodomys brandtii*). Front. Physiol..

[81] Landen J.G., Vandendoren M., Killmer S., Bedford N.L., Nelson A.C. (2024). Huddling substates in mice facilitate dynamic changes in body temperature and are modulated by Shank3b and Trpm8 mutation. Commun. Biol..

[82] Brittebo E.B. (1995). Metabolism-dependent toxicity of methimazole in the olfactory nasal mucosa. Pharmacol. Toxicol..

[83] Genter M.B., Deamer N.J., Blake B.L., Wesley D.S., Levi P.E. (1995). Olfactory toxicity of methimazole: Dose-response and structure-activity studies and characterization of flavin-containing monooxygenase activity in the Long-Evans rat olfactory mucosa. Toxicol. Pathol..

[84] Greer D., Lei T., Kryshtal A., Jessen Z.F., Schwartz G.W. (2025). Visual identification of conspecifics shapes social behavior in mice. Curr. Biol..

[85] Harshaw C., Kojima S., Wellman C.L., Demas G.E., Morrow A.L., Taft D.H., Kenkel W.M., Leffel J.K., Alberts J.R. (2022). Maternal antibiotics disrupt microbiome, behavior, and temperature regulation in unexposed infant mice. Dev. Psychobiol..

[86] Han Y., Ai L., Sha S., Zhou J., Fu H., Sun C., Liu R., Li A., Cao J.L., Hu A. (2024). The functional role of the visual and olfactory modalities in the development of socially transferred mechanical hypersensitivity in male C57BL/6J mice. Physiol. Behav..

