# Supplementary material for: Olfactory Disruption Restructures Collective Behavior and Increases Cohesive Group Dynamics
Source: Biology (Basel). 2026 Feb 20;15(4):360. doi: 10.3390/biology15040360 (PMC12938050; doi:10.3390/biology15040360)
Supplement: Supplementary file 1 [file biology-15-00360-s001.zip › supplementary materials/Chen et al. 2026 Biology Supplementary Figures Proofread.docx]

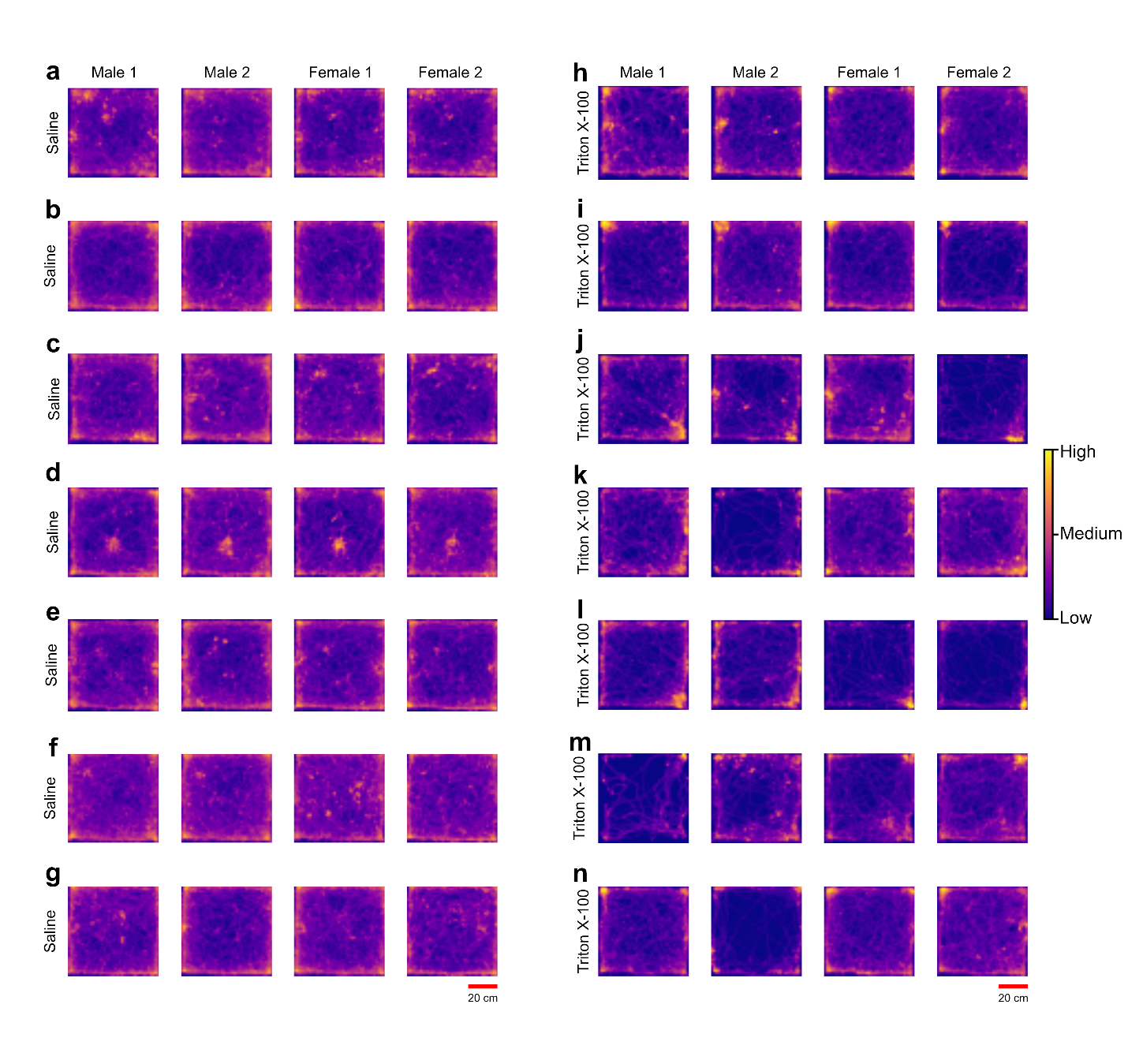


**Figure S1. Spatial analysis of individual animal occupancy for Triton X-100 experiments.**

(**a-g**) Occupancy maps showing the spatial distributions of each animal irrigated with saline. Each sub panel represents one recording.

(**h-n**) As in **a**, but for mice irrigated with Triton X-100.


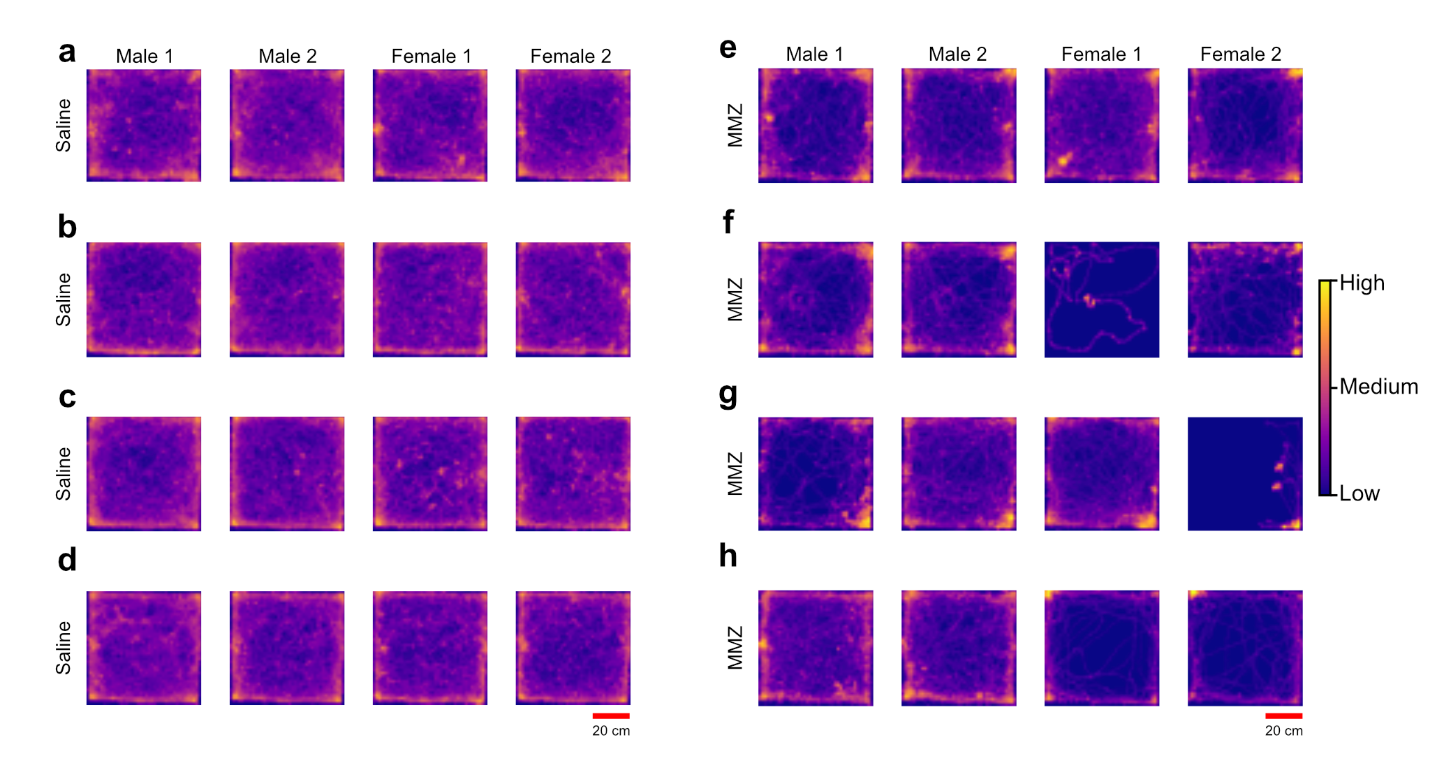


**Figure S2. Spatial analysis of individual animal occupancy for MMZ experiments.**

(**a-d**) Occupancy maps showing the spatial distributions of each animal injected with saline. Each sub panel represents one recording.

(**e-h**): As in **a**, but for mice injected with MMZ.


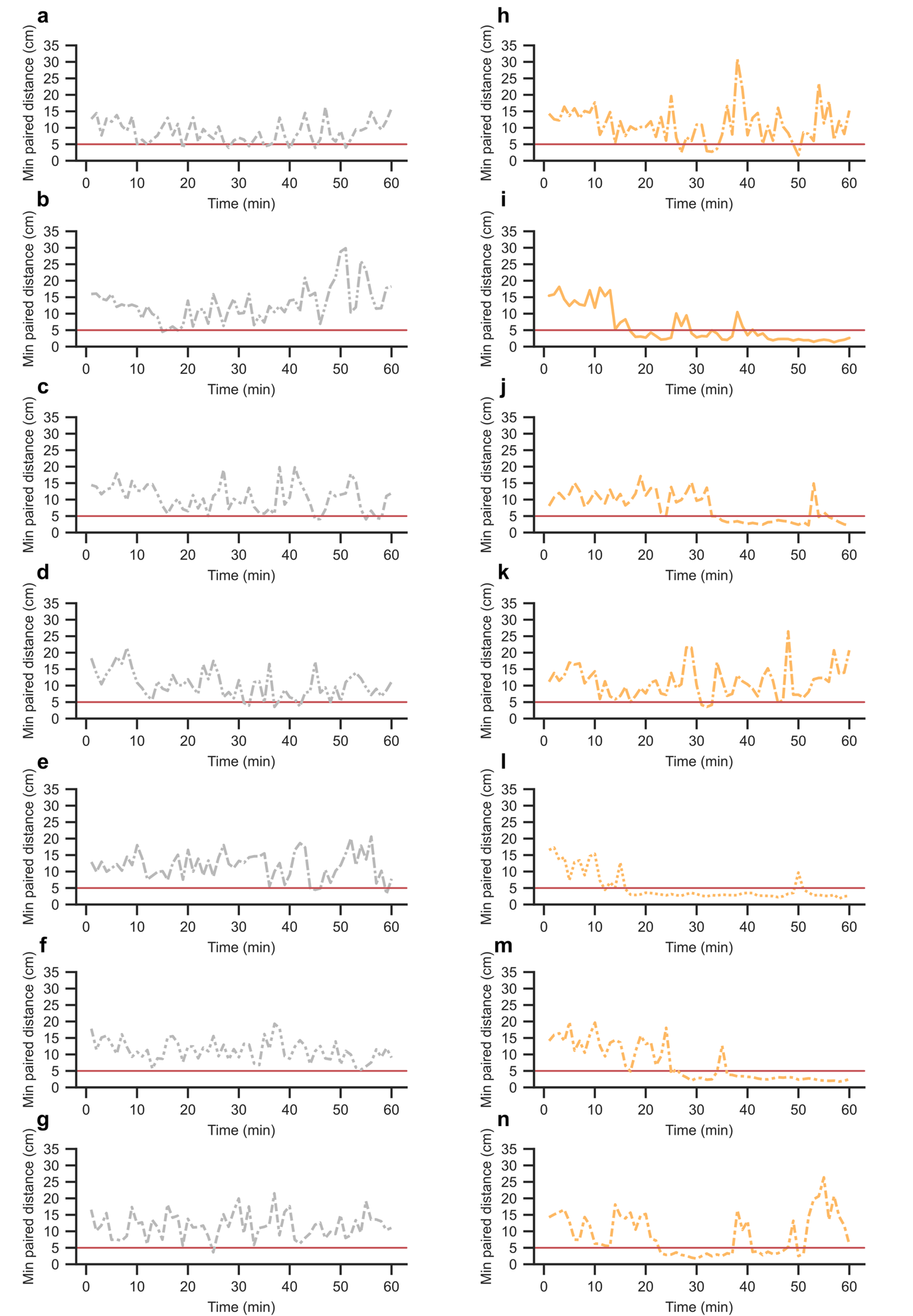


**Figure S3. Minimum dyadic distances for Triton X-100 experiments.**

(**a-g**) Minimum dyadic distance, calculated as the smallest of the six pairwise distances between mice, binned by one-minute intervals across the first hour of the recording. Each sub panel represents one recording from mice irrigated with saline, corresponding to Fig S1. The red line marks the 5 cm threshold used as the spatial criterion for sustained proximity.

(**h-n**) As in **a**, but for mice irrigated with Triton X-100.


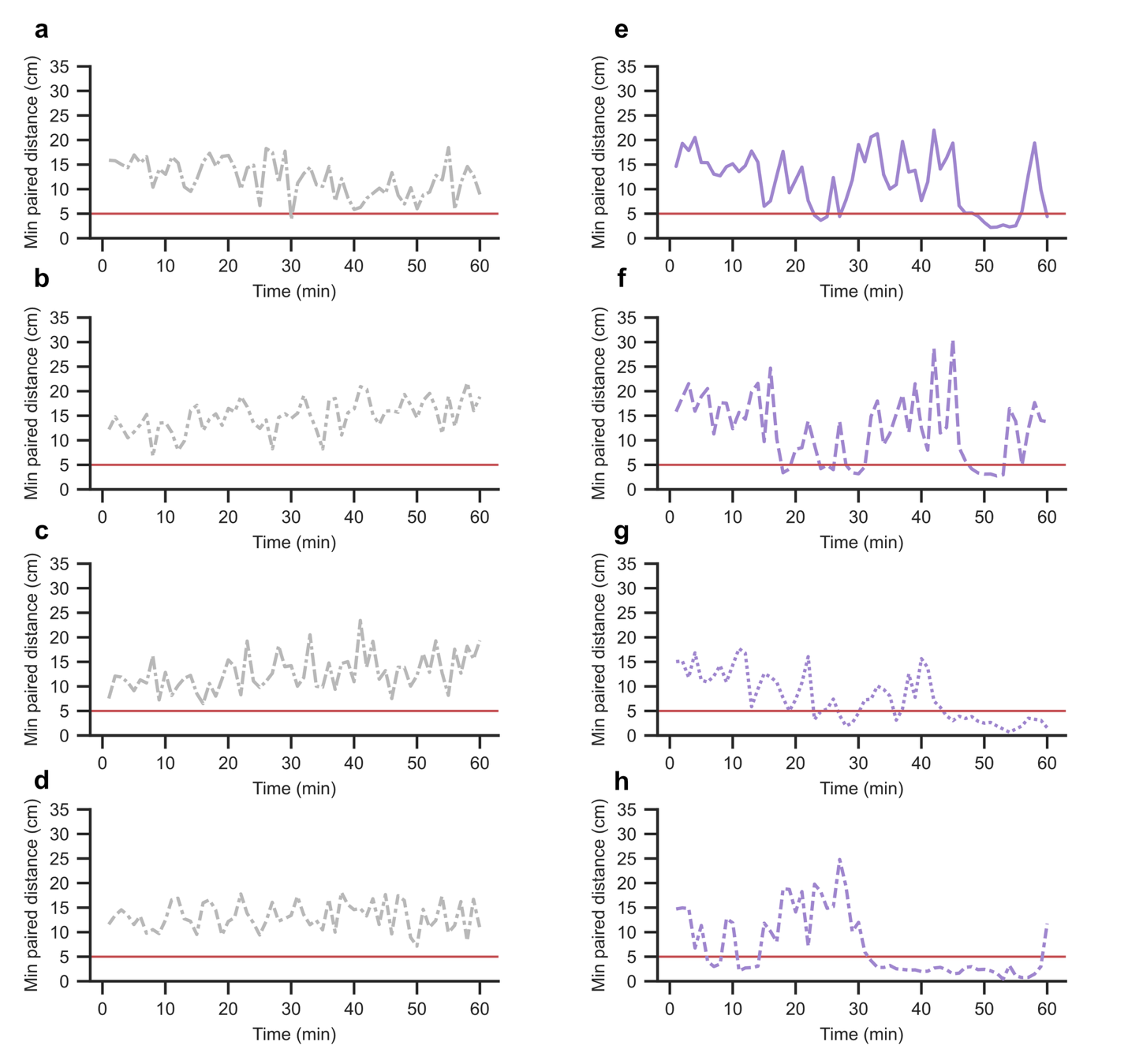
**Figure S4. Minimum dyadic distances for MMZ experiments.**

(**a-d**) Minimum dyadic distance, calculated as the smallest of the six pairwise distances between mice, binned by one-minute intervals across the first hour of the recording. Each sub panel represents one recording from mice injected with saline, corresponding to Fig S2. The red line marks the 5 cm threshold used as the spatial criterion for sustained proximity.

(**e-h**) As in **a**, but for mice injected with MMZ.


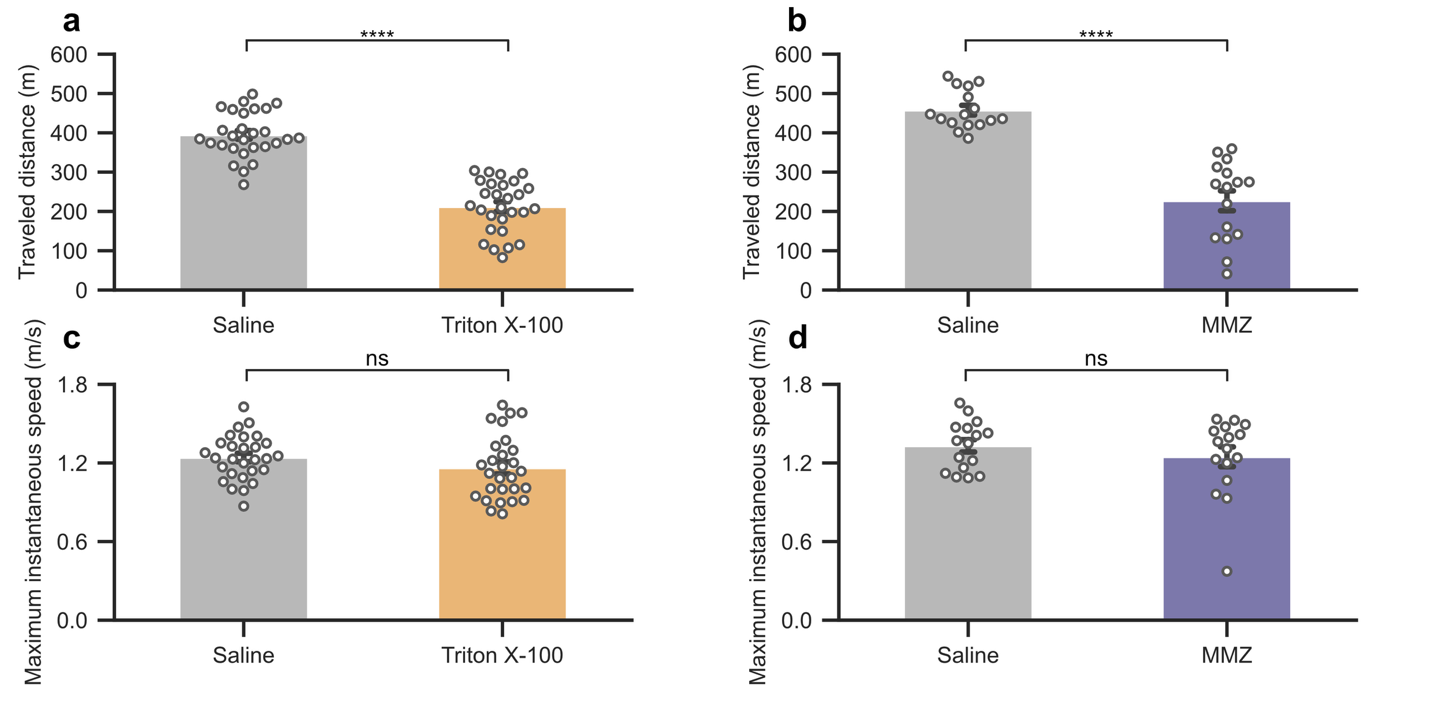


**Figure S5. Olfactory-impaired mice showed reduced locomotor activities, but not ability to move.**

(**a**) Total distance traveled in the first hour for mice irrigated with saline (mean = 394.8, SE = 10.9) or Triton X-100 (mean = 212.1, SE = 12.5). A linear mixed-effects model was fitted with condition as a fixed effect and recording group as a random intercept. The effect of condition was highly significant (β = -182.7, SE = 16.6, t = -11.0, *p* < 0.0001), indicating reduced travel distance in the Triton X-100 condition.

(**b**) As in **a**, but for mice injected with saline (mean = 457.7, SE = 12.3) or MMZ (mean = 227.0, SE = 25.2). A linear mixed-effects model was fitted with condition as a fixed effect and recording group as a random intercept. The effect of condition was highly significant (β = -230.6, SE = 28.1, t = -8.2, *p* < 0.0001), indicating reduced travel distance in the MMZ condition.

(**c**) The maximum speed in the first hour for mice irrigated with saline (mean = 1.24, SE = 0.03) or Triton X-100 (mean = 1.16, SE = 0.05). A linear mixed-effects model was fitted with condition as a fixed effect and recording group as a random intercept. The effect of condition was not significant (β = -0.08, SE = 0.07, t = -1.19, p = 0.26).

(**d**) As in **c**, but for mice injected with saline (mean = 1.33, SE = 0.05) or MMZ (mean = 1.25, SE = 0.08). A linear mixed-effects model was fitted with condition as a fixed effect and recording group as a random intercept. The effect of condition was not significant (β = -0.08, SE = 0.10, t = -0.81, p = 0.45).


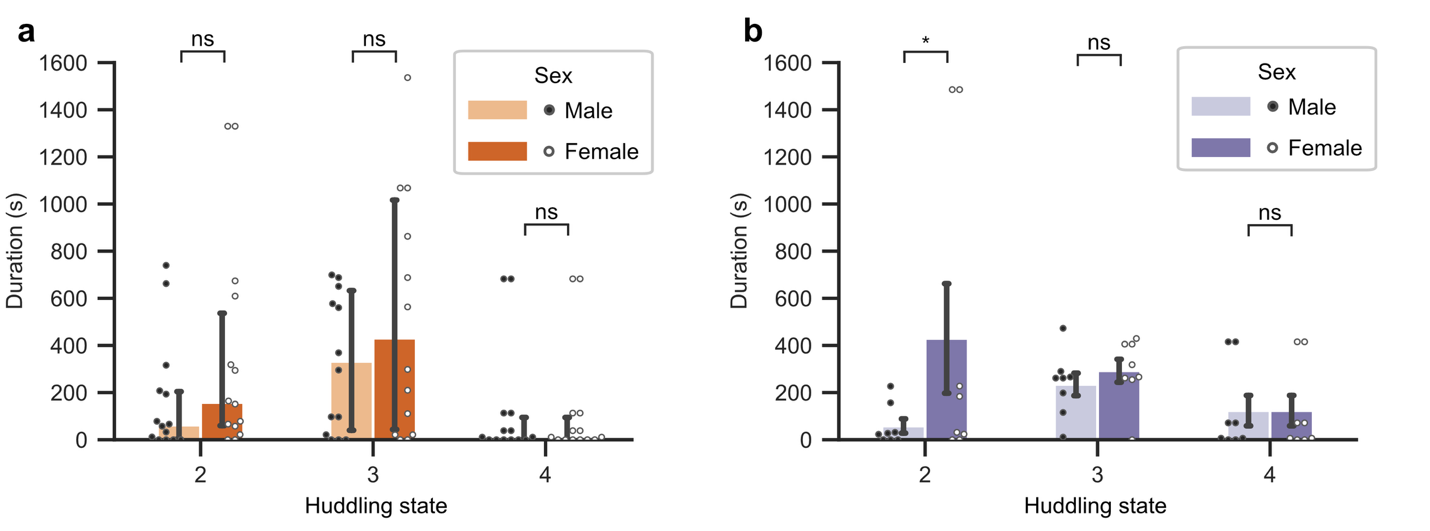


**Fig S6. Both male and female olfactory-impaired mice exhibit huddling behavior.**
(**a**) Total duration spent huddling across different huddling states, separated by sex, for mice irrigated with Triton X-100. State 2 corresponds to dyadic huddling, state 3 to three-mouse huddling, and state 4 to four-mouse huddling. A generalized linear mixed-effects model with a Gamma distribution and log link was fitted, with sex and huddling state as fixed effects and recording group and animal as random effects. No main effect of sex was observed (β = 0.20, SE = 0.93, t = 0.22, p = 0.83). For dyadic huddling, males had a median duration of 61.78 s (IQR = 201.25 s) and females 157.82 s (IQR = 476.94 s). For three-mouse huddling, male median = 332.18 s (IQR = 592.39 s) and female median = 430.75 s (IQR = 973.24 s). For four-mouse huddling, male and female medians were 10.87 s (IQR = 9.34 s).

(**b**) As in (**a**), but for mice treated with MMZ. A linear mixed-effects model was fitted with sex and huddling state as fixed effects and recording group and animal as random effects. A significant main effect of sex was observed (β = 371.54, SE = 142.27, t = 2.61, p = 0.013), driven by greater time spent by females in the dyadic huddling state (females: mean = 429.66 s, SE = 232.35; males: mean = 58.12 s, SE = 30.25). No significant sex differences were observed in the three- or four-mouse huddling states. All multiple comparisons were corrected using Holm’s method.
